# Development of ELISAs for diagnosis of acute typhoid fever in Nigerian children

**DOI:** 10.1371/journal.pntd.0005679

**Published:** 2017-06-22

**Authors:** Jiin Felgner, Aarti Jain, Rie Nakajima, Li Liang, Algis Jasinskas, Eduardo Gotuzzo, Joseph M. Vinetz, Fabio Miyajima, Munir Pirmohamed, Fatimah Hassan-Hanga, Dominic Umoru, Binta Wudil Jibir, Safiya Gambo, Kudirat Olateju, Philip L. Felgner, Stephen Obaro, D. Huw Davies

**Affiliations:** 1 Division of Infectious Diseases, School of Medicine, University of California Irvine, Irvine, California, United States of America; 2 Alexander von Humboldt Institute of Tropical Medicine, Universidad Peruana Cayetano Heredia, Lima, Peru; 3 Hospital Nacional Cayetano Heredia, Lima, Peru; 4 Division of Infectious Diseases, Department of Medicine, University of California San Diego, La Jolla, California, United States of America; 5 Department of Molecular and Clinical Pharmacology, University of Liverpool, Liverpool, United Kingdom; 6 Department of Pediatrics, Aminu Kano Teaching Hospital, Kano, Nigeria; 7 Maitama District Hospital, Abuja, Nigeria; 8 Hasiya Bayero Pediatric Hospital, Kano, Nigeria; 9 Department of Pediatrics, Murtala Specialist Hospital, Kano, Nigeria; 10 University of Abuja Teaching Hospital, Gwagwalada, Nigeria; 11 Department of Pediatrics, University of Nebraska Medical Center, Omaha, Nebraska, United States of America; 12 International Foundation against Infectious Diseases in Nigeria (IFAIN), Abuja, Nigeria; Oxford University Clinical Research Unit, VIET NAM

## Abstract

Improved serodiagnostic tests for typhoid fever (TF) are needed for surveillance, to facilitate patient management, curb antibiotic resistance, and inform public health programs. To address this need, IgA, IgM and IgG ELISAs using *Salmonella enterica* serovar Typhi (*S*. Typhi) lipopolysaccharide (LPS) and hemolysin E (t1477) protein were conducted on 86 Nigerian pediatric TF and 29 non-typhoidal *Salmonella* (NTS) cases, 178 culture-negative febrile cases, 28 “other” (i.e., non-*Salmonella*) pediatric infections, and 48 healthy Nigerian children. The best discrimination was achieved between TF and healthy children. LPS-specific IgA and IgM provided receiver operator characteristic areas under the curve (ROC AUC) values of 0.963 and 0.968, respectively, and 0.978 for IgA+M combined. Similar performance was achieved with t1477-specific IgA and IgM (0.968 and 0.968, respectively; 0.976 combined). IgG against LPS and t1477 was less accurate for discriminating these groups, possibly as a consequence of previous exposure, although ROC AUC values were still high (0.928 and 0.932, respectively). Importantly, discrimination between TF and children with other infections was maintained by LPS-specific IgA and IgM (AUC = 0.903 and 0.934, respectively; 0.938 combined), and slightly reduced for IgG (0.909), while t1477-specific IgG performed best (0.914). A similar pattern was seen when comparing TF with other infections from outside Nigeria. The t1477 may be recognized by cross-reactive antibodies from other acute infections, although a robust IgG response may provide some diagnostic utility in populations where incidence of other infections is low, such as in children. The data are consistent with IgA and IgM against *S*. Typhi LPS being specific markers of acute TF.

## Author summary

In many African countries, clinical management of children that present with symptoms of bacterial sepsis, such as typhoid fever (TF) caused by *Salmonella* Typhi, consists of empiric broad spectrum antibiotics. Blood culture remains the gold-standard for diagnosis, but is slow, suffers from poor sensitivity, and often unavailable. Consequently multi-drug resistant bacteria have emerged that are difficult to manage with antibiotics. There is an urgent need to develop rapid, sensitive and affordable tests for patient diagnosis, help curb antibiotic resistance, and inform public health preventive strategies such as the deployment of vaccines. Here, we have assessed antibodies to *S*. Typhi lipopolysaccharide (LPS) and hemolysin E (HylE, t1477) in the sera of Nigerian children with acute TF and compared them with heathy children, children with other febrile infections, and adults from around the world with a variety of other bacterial infections. The key finding concerns LPS. This is a common cell-wall component present in many bacterial species. Yet despite this, *S*. Typhi LPS-specific IgA and IgM are excellent markers of acute TF in Nigerian children, and insensitive to other non-salmonelloses. This surprising finding suggests a rapid point-of-care test for TF can be developed based on detection of LPS-specific IgA+IgM.

## Introduction

Salmonelloses are a group of potentially fatal bacteremias caused by different serovars of *Salmonella enterica*. Typhoid fever (TF), caused by the human-specific serovar *S*. Typhi, is a global health problem, especially in developing countries [1, 2]. In 2010 there were an estimated 26.9 million TF episodes worldwide, with a case-fatality rate of ~ 1% [3]. Salmonellosis caused by nontyphoid *Salmonella* (NTS) serovars are caused predominantly by the zoonotic serovars, *S*. Typhimurium and *S*. Enteritidis [4–7]. These are emerging in sub-Saharan Africa as an important cause of bacteremia in young children, typically when associated with malnutrition, malaria, severe anemia, and/or HIV co-infection [6, 8–11]. Case-fatality rates for blood-borne, or invasive, NTS (iNTS) infection is higher than that for typhoid, typically ~20% [4, 6, 12], although the antibiotic treatment regimen is the same. Over-use of empiric broad-spectrum antibiotic treatment for undifferentiated febrile disease has led to an increase in antibiotic resistance in both typhoidal and nontyphoidal serovars, and the potential for new antibiotics is not encouraging [1, 13, 14]. The development of effective vaccines to prevent invasive salmonellosis is therefore an important global health priority [1, 15]

Accurate diagnosis of salmonellosis remains a challenge in endemic settings. Clinically, initial presentation with typhoid or NTS disease is usually with non-differentiating fever alone, and often without symptoms of gastroenteritis that would indicate a *Salmonella* infection [7]. Bacterial culture is the gold standard for diagnosis of both typhoid and iNTS disease. However, culture suffers from poor sensitivity, and culture facilities are very limited in resource poor settings such as Nigeria and other countries in Africa. Even when such facilities are available, the time to a laboratory diagnosis is around 48 hours, and is often unaffordable for most patients. An inactivated-*Salmonella* agglutination test, developed by Widal >100 years ago, is a rapid and affordable single-step test. It remains the mainstay of diagnosis in many developing countries, even when culture facilities are available. However, the Widal’s test has poor specificity, thought to be caused by antigens shared between *S*. *enterica* serovars, and between other species of bacteria, such as *Brucella melitnesis* [16]. The Widal’s test also fails to discriminate between current and previous exposure, thus requiring two samples to be taken 7–10 days apart to monitor for an increase in titer. In practice, the decision to treat with antibiotics has to be made on the basis of the first test, and confirmatory convalescent testing is often not practicable or irrelevant for immediate patient management. It is also less sensitive in the acute stage of infection when IgG titers are lower.

The lack of accurate tests for surveillance also has resulted in only limited understanding of epidemiology of salmonellosis in Africa. The high mortality, particularly in children with iNTS infections, and the recent emergence of drug resistance, emphasize the need for a better understanding of the epidemiology before the rational design and implementation of control measures, including vaccines, can be effectively deployed.

In this study we have addressed the development of improved serodiagnostics with well-defined serum samples collected from febrile children in Nigeria. Based on proteome microarray screening data published recently [17], we hypothesized that LPS and/or the hemolysin E (HylE, t1477) antigen may have diagnostic utility for TF. However, it was unknown from the original study if these antigens were cross-reactive for other bacteremias. Here we have evaluated IgG, IgM and IgA ELISAs using purified *S*. Typhi LPS and HlyE using culture-confirmed pediatric bacteremias, including typhoid, iNTS disease, and ‘other’ febrile diseases, as well as healthy Nigerian children, and healthy adults from the U.S. We find LPS-specific IgA and IgA+M ELISA, in particular, are sensitive in diagnosing acute typhoid in these children, and descriminate well between typhoid and healthy, and other febrile bacteremias commonly encountered in Nigeria.

## Results

### Pilot study with multi-LPS microarray

**Fig 1:**
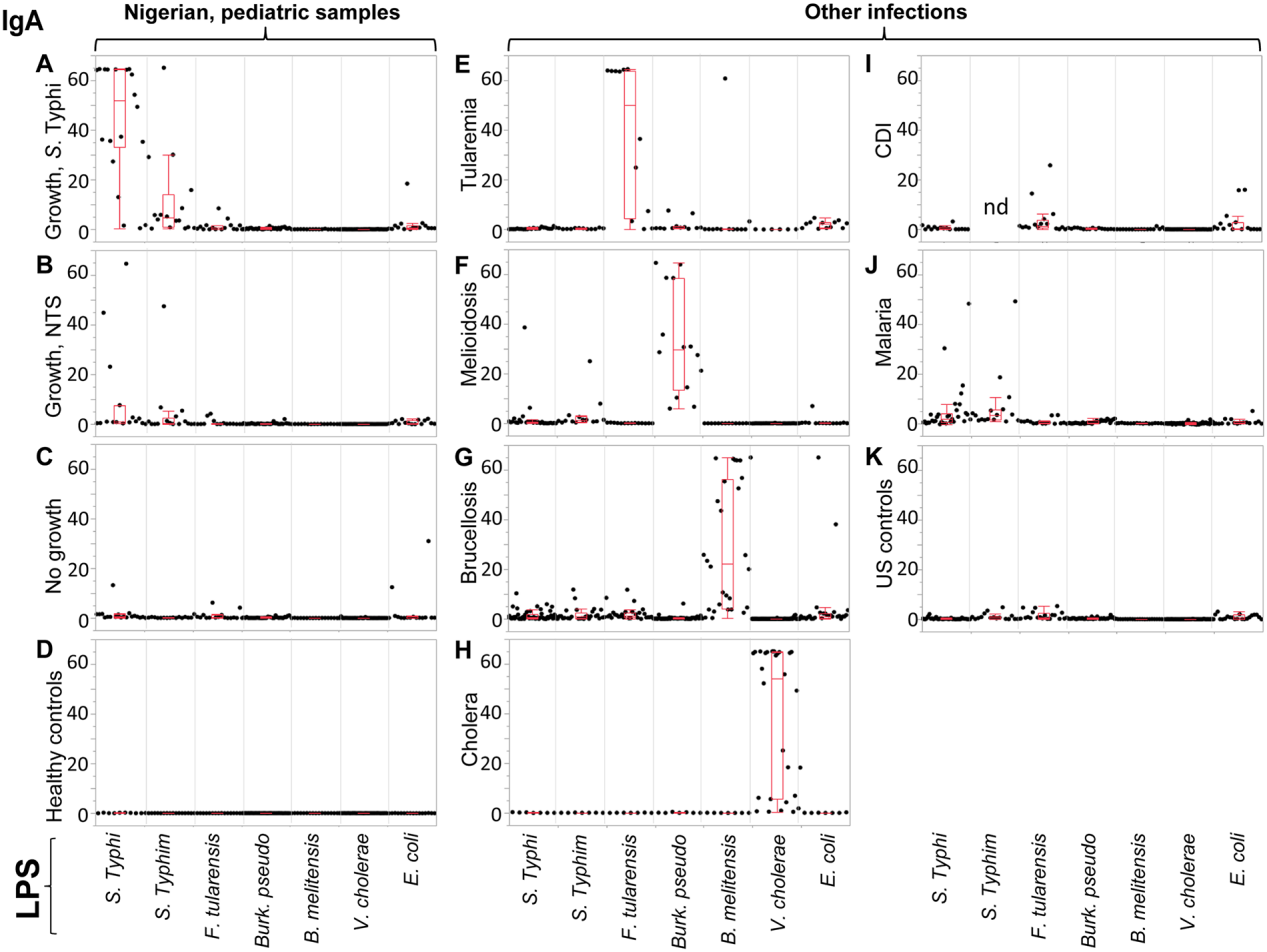
Pilot study with LPS microarray, IgA data. Box plots of IgA antibody signals (y-axis, mean pixel intensity x10^3^) from different groups reacting to LPS from different organisms (listed along x-axis). Dots represent individual serum samples. (A) Nigerian pediatric culture-positive typhoid. (B) Nigerian pediatric culture-positive NTS. (C) Nigerian pediatric febrile cases, culture negative. (D) Nigerian pediatric healthy controls. (E) Spanish adult tularemia. (F) Thai adult melioidosis. (G) Peruvian adult brucellosis. (H) Bangladeshi adult cholera. (I) British adult *C*. *difficile* infections (CDI). (J) Kenian, Malian and Papua New Guinean adult malaria. (K) U.S. healthy adult controls. NTS, non-typhoid *Salmonella*; nd, not done.

**Fig 2:**
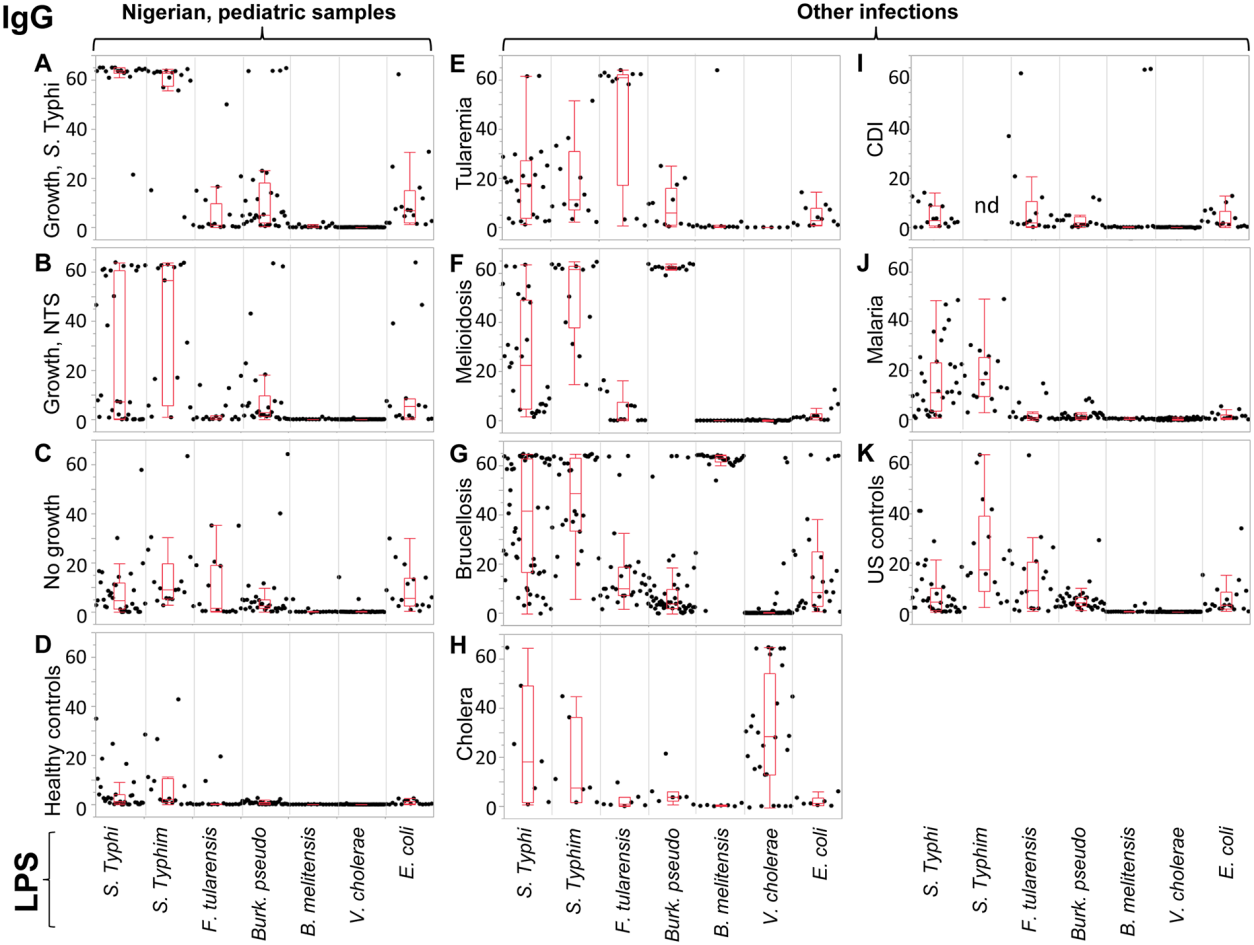
Pilot study with LPS microarray, IgG data. Box plots of IgG antibody signals (y-axis, mean pixel intensity x10^3^) from different groups reacting to LPS from different organisms (listed along x-axis). Dots represent individual serum samples. Panels (A) to (K) as Fig 1. NTS, non-typhoid *Salmonella*; nd, not done.

In a previous study [17] we confirmed the potential utility of *S*. Typhi LPS-specific IgA for the diagnosis of acute typhoid in sera from Nigerian children. Before pursuing this further, we wished to investigate potential cross-recognition of LPS by antibodies from other acute infections that might lead to a false-positive diagnosis of typhoid. For this we conducted a pilot study using a microarray displaying LPS from 7 different bacterial pathogens (*S*. Typhi, *S*. Typhimurium, *F*. *tularensis*, *B*. *pseudomallei*, *B*. *melitensis*, *V*. *cholerae* and *E*. *coli*), and probed it with sera from Nigerian pediatric samples and adult samples available from other febrile diseases, and controls. The data for IgA and IgG reactivity are summarized in the box plots in Figs 1 and 2, respectively. Panels A-D show Nigerian pediatric samples. As reported previously, IgA reactivity for *S*. Typhi LPS was strongest in typhoid cases (N = 16; Fig 1A), largely absent from ‘No Growth’ (N = 16; Fig 1C) and healthy control (N = 16; Fig 1D) samples, while present in a few individuals with culture-confirmed NTS (N = 16; Fig 1B) presumably owing to the antigenic similarities between LPS from related *Salmonella* serovars. Although there is a range of signals from the typhoid cases, only one sample was negative. We then examined the reactivity of sera from other bacteremias for other locations outside Nigeria, as follows: tularemia from Spain (N = 12; Fig 1E), melioidosis from Thailand (N = 7 acute, and N = 7 convalescent; Fig 1F), brucellosis from Peru (N = 12 acute, and N = 16 convalescent; Fig 1G), cholera from Bangladesh (N = 7 acute, and N = 7 convalescent; Fig 1H), and *C*. *difficile* infections (CDI) from the UK (N = 16; Fig 1I). Also probed were malaria samples from Mali, PNG and Kenya (N = 16; Fig 1J) and healthy controls from the U.S. (N = 20; Fig 1K). With the exception of two melioidosis cases and two malaria cases, IgA from these other infections did not cross-react with *S*. Typhi or *S*. Typhimurium LPS in this study.

Of note, IgA from other gram negative bacteremias did recognize the LPS appropriate to the infecting organism. Thus, IgA in individuals with acute tularemia specifically recognized *F*. *tularensis* LPS (Fig 1E), melioidosis IgA specifically recognized *B*. *pseudomallei* LPS (Fig 1F), brucellosis IgA specifically recognized LPS from *B*. *melitensis* (Fig 1G) and cholera IgA specifically recognized *V. cholerae* LPS (Fig 1H). This shows the lack of cross-reactivity for *S*. Typhi LPS was not due to a lack of anti-LPS antibodies in these other infections. In several cases, the signal intensity of the LPS-specific IgA response correlated with stage of infection. For example, of the 12 tularemia samples, the six 2nd time-point samples after MA seroconversion (late acute stage) gave maximal signals against *F*. *tularensis* LPS, while the remaining samples were the 1st time point prior to seroconversion (early acute).

For IgG, the most robust signals against *S*. Typhi and *S*. Typhimurium LPS were from the Nigerian acute typhoid cases. However, IgG was not a reliable marker of acute typhoid in Nigerian children in this array study. IgG against these antigens were particularly common among all the samples tested, but particularly in the Thai melioidosis, Peruvian brucellosis, malaria samples from various locations (Fig 2F, 2G and 2J, respectively), and the US negative controls (Fig 2K), consistent with the exposure to *Salmonella* species being widespread globally. These data are consistent with IgG being associated with both acute and convalescent (previous) exposure. Indeed, most individuals tested had IgG to multiple LPS species. For example, many of the Nigerian individuals in panels A-D also have LPS-specific IgG to *E*. *coli* and *B*. *pseudomallei*, which may indicate a previous exposure to these organisms and/or cross-reactivity from other infections. Antibodies against *F*. *tularenisis* are also quite common among different populations where tularemia is non-endemic (e.g., U.S), and may reflect cross-recognition of antibodies to other non-pathogenic *Francisella* species [18].

### Optimization of ELISAs

The data from the pilot study described above indicated that LPS-specific IgA may have utility for discriminating between acute and convalescent typhoid or other acute infections. While a more deployable array format is currently under development [19], in parallel we decided to develop an ELISA test for typhoid. The ELISA is inexpensive, robust and provides results more quickly than blood culture. Two batches of HlyE were used in the course of this study which, when compared by ELISA using all 349 Nigerian samples correlated with an r^2^ = 0.922 and a slope = 1.05 using Spearmann’s rank correlation (S1 Fig). The optimal conditions for ELISAs were initially determined using individual serum samples from three Nigerian typhoid patients and a healthy control. The optimized concentrations of the coating antigens were determined by titration to be 1.25μg/ml for LPS, and 2.5μg/ml for HlyE (t1477). Two serum dilutions, 1/100 and 1/200, were evaluated for the highest ratio when comparing heathy controls and culture-confirmed typhoid. For t1477 ELISA, 1/100 was selected, while for LPS ELISA, 1/200 was found to give the higher ratio and selected for subsequent studies. Two secondary antibody dilutions recommended by the manufacturer, 1/12,500 and 1/25,000, were evaluated for optimal signal to background ratio. For IgA and IgM ELISA, 1/12500 dilution was selected, while for IgG ELISA 1/25000 dilution was used. Once established, a standard operating procedure was used throughout the study. Batches of ELISA plates were prepared by pre-coating plates with antigen, blocking, and then storing dried at 4°C in desiccated pouches until required for use.

### Detection of LPS-specific antibodies in serum samples by ELISA

A total of 495 serum samples were used (Table 1) and tested for LPS-specific IgG, IgA, IgM and IgA+IgM in separate ELISAs. The samples comprised 369 Nigerian pediatric samples, consisting of culture-confirmed typhoid (“*S*. Typhi”, n = 86), non-typhoid *Salmonella* (“NTS”) disease (n = 29) or other bacteremias (“Other”, n = 28; listed in Table 2), as well as febrile cases that were blood culture-negative for any bacteria (“No Growth”, n = 178), and healthy Nigerian control children (“Healthy”, n = 48). Also tested by ELISA were well-defined sera from tularemia, brucellosis, and malaria cases, as well as U.S. controls.

**Table 1:** Sera samples used to probe LPS array and ELISAs.

Samples probed		Country	LPS array (sample N)	ELISA (sample N)
**Nigerian children**	Febrile, Growth, *S*. Typhi (*Salmonella* Typhi)	Nigeria	16	86
	Febrile, Growth, NTS ^a^	Nigeria	16	29
	Febrile, Growth, Other (see Table 2)	Nigeria	nd ^b^	28
	Febrile, No Growth	Nigeria	16	178
	Healthy Nigerian controls	Nigeria	16	48
**Other bacteremia**	Tularemia (*Francisella tularensis*), early acute and late acute	Spain	6 each	12
	Brucellosis (*Brucella melitensis*), acute and convalescent	Peru	12 and 16	16
	Melioidosis (*Burkholderia pseudomallei*), acute and convalescent	Thailand	7 each	nd ^b^
	Cholera (*Vibrio cholerae*), acute and convalescent	Bangladesh	7 each	nd ^b^
	CDI colitis (*Clostridium difficile*), acute	UK	16	nd ^b^
**Other infections**	Malaria parasitemia (*Plasmodium falciparum*)	Papua New Guinea, Mali, Kenya	16	48
**Other controls**	Healthy US adults	USA	20	50

^a^ Non-typhoidal *Salmonella*

^b^nd, not done

**Table 2:** List of non-*Salmonella* Nigerian bacteremias confirmed by blood culture assayed by ELISA.

	number samples
*Streptococcus pneumoniae*	1
*Staphylococcus aureus*	4
*Pseudomonas* sp.	3
*Pseudomonas aeruginosa*	3
*Neisseria meningitidis*	1
*Klebsiella terrigena*	1
*Klebsiella pneumoniae*	1
*Haemophilus influenzae*	1
*Escherichia coli*	2
*Enterococcus faecalis*	4
*Enterobacter cloacae*	2
Coagulate negative *Staphylococcus*	2
Alpha haemolytic *Streptococcus*	2
*Acinetobacter* sp.	1

**Fig 3:**
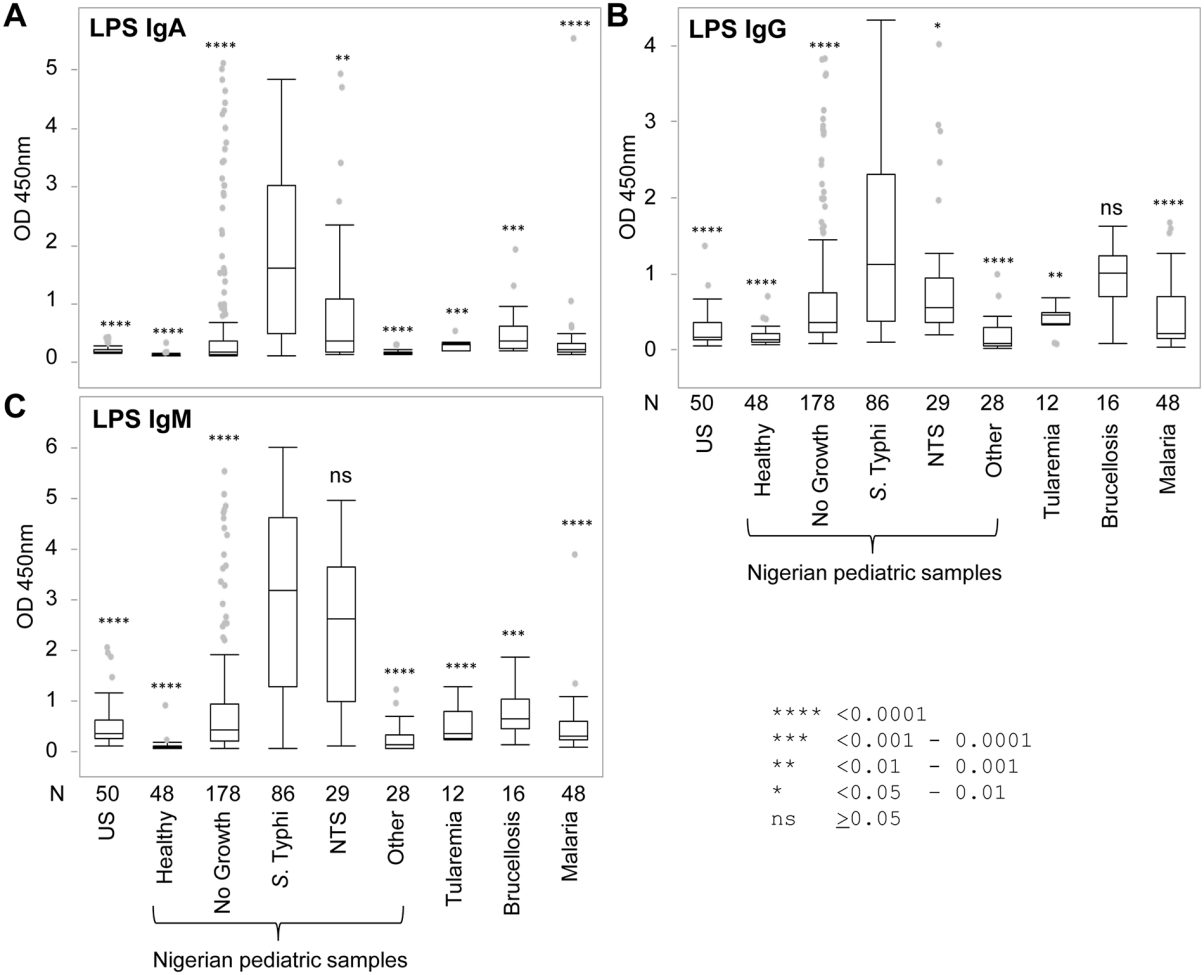
Reactivity of different groups to LPS. Box plots of LPS ELISA data using sera from different pediatric groups from Nigeria, ‘other’ infections, and healthy US controls (see Methods for details). The plots show OD 450 nm for (A) IgA, (B) IgG, and (C) IgM. Sample size N shown below the x-axis. Box plots represent 3rd and 1st quartiles with the median line within the box; the whiskers indicate + and − 1.5*(interquartile range), and dots show outliers. Asterisks above box plots represent *P*-values of nonparametric comparisons between the *S*. Typhi group and each of the other groups according to the key, using the Wilcoxon method; ns, not significant.

Results of all ELISAs are summarized as box plots in Fig 3; the same data are also presented as bar charts in Supporting Information S2 Fig. In IgA ELISAs, the median OD value of the typhoid group was statistically different from all other groups when tested by the Wilcoxon method. Of these other groups, the NTS disease group showed the highest reactivity, due presumably to antibodies to LPS from NTS serovars cross-reacting with LPS from *S*. Typhi. While the difference between the medians of the *S*. Typhi and “No Growth” groups were highly significant (*P* < 0.0001), the latter group contained a large number of outliers that may correspond to blood culture false-negatives. Significantly, none of the 48 healthy Nigerian controls or 28 Nigerian “other” infections were seropositive for LPS IgA. Reactivity by non-Nigerian other infection groups was generally very low, although some Peruvian brucellosis cases had reactivity or cross-reactivity to *S*. Typhi LPS. There were two outliers in the malaria group (N = 48) with an LPS-IgA response. Although it is not known whether these individuals had a co-infection with *Salmonella*, association of malaria with salmonellosis is well known to the medical community. The IgG response to LPS (Fig 3B) was elevated in all groups, consistent with widespread previous exposure to *Salmonella* sp. The *P*-values for NTS, tularemia and brucellosis were larger than for IgA, with brucellosis failing to reach significance. Overall, the pattern of reactivity by IgM to LPS (Fig 3C) was similar to that of IgA, with the notable exception of NTS which was not significantly different to typhoid cases. As for IgA, IgM reactivity in healthy Nigerian children was very low, whereas IgM reactivity by the ‘other’ infections from Nigeria and elsewhere were overall higher than for IgA. Overall, the data indicate that the LPS-specific IgA has the best potential of the three isotypes for the diagnosis of acute typhoid from other febrile diseases.

### Detection of t1477-specific antibodies in serum samples by ELISA

**Fig 4:**
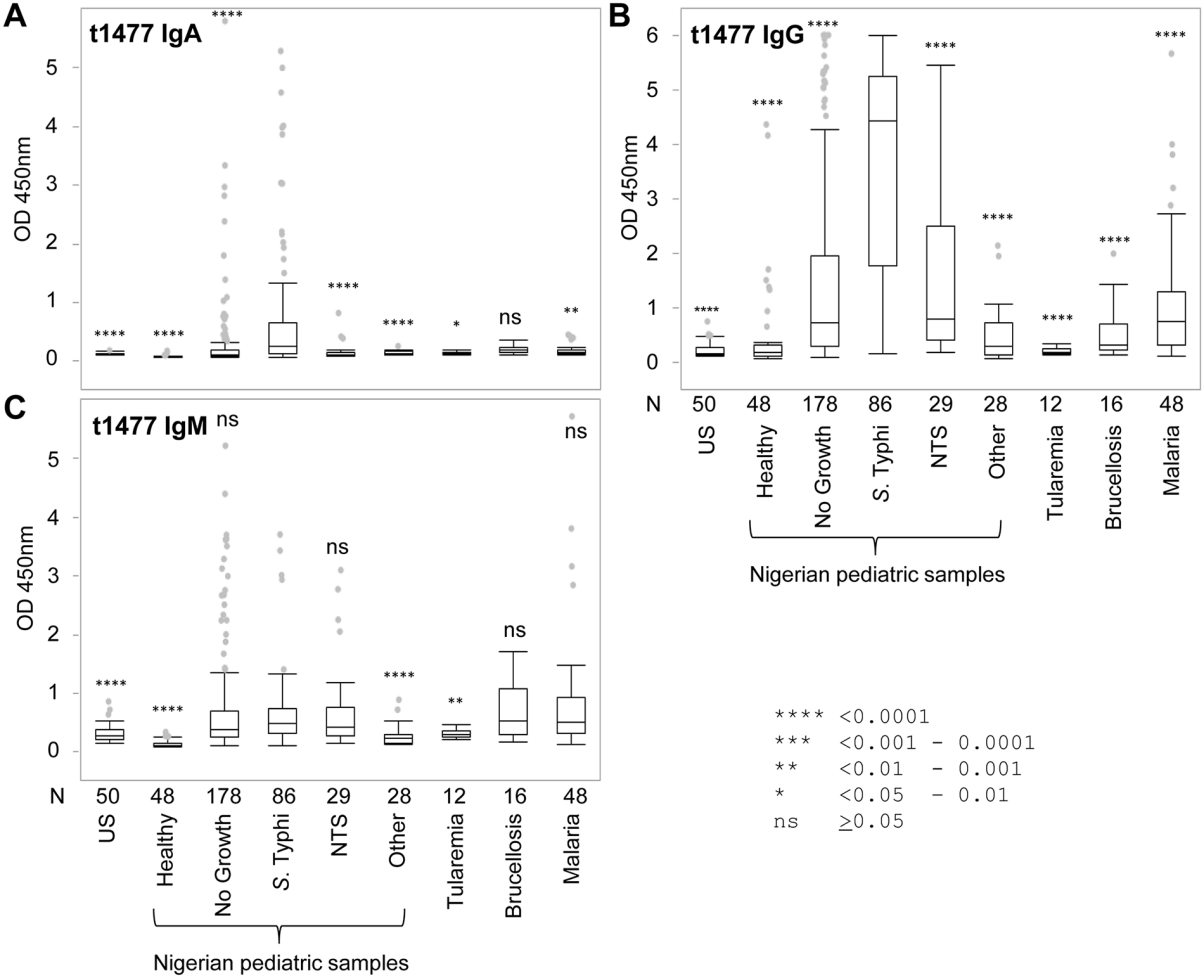
Reactivity of different groups to hemolysin E (t1477). Box plots of t1477 ELISA data using sera from different pediatric groups from Nigeria, ‘other’ infections, and healthy US controls (see Methods for details). The plots show OD 450 nm for (A) IgA, (B) IgG, and (C) IgM. Sample size N shown below the x-axis. Box plots represent 3rd and 1st quartiles with the median line within the box; the whiskers indicate + and − 1.5*(interquartile range), and dots show outliers. Asterisks above box plots represent *P*-values of nonparametric comparisons between the *S*. Typhi group and each of the other groups according to the key, using the Wilcoxon method; ns, not significant.

Previous experiments using a *S*. Typhi full proteome array [17] revealed very few protein antigens with utility for diagnosing typhoid fever in Nigerian children. However, the hemolysin E protein (HylE, t1477) did emerge as a potential candidate, and is examined further here and in the following section for sensitivity and specificity using the full serum collection (N = 495 as described for LPS above) by ELISA for IgA, IgG and IgM (Fig 4). The same data are also presented as bar charts in Supporting Information S3 Fig. Overall, IgA reactivity was low among all the groups. Nevertheless, the ‘*S*. Typhi’ and ‘No Growth’ groups had the largest number of seropositive individuals (Fig 4A). IgA-responses to t1477 provided better discrimination between ‘S. Typhi’ and ‘NTS’ groups, although sensitivity of detection in both groups was low (detailed in the next section). By comparison, the IgG response to t1477 was elevated in all groups (Fig 4B). The highest median IgG signal was seen in the pediatric typhoid group, with the ‘No growth’ and ‘NTS’ groups having the next highest signals overall. Interestingly the Nigerian healthy children were the lowest of the Nigerian groups, although there were a number of outliers with IgG signals. IgG alone does not allow discrimination between ongoing or previous episodes of typhoid, although the negligible reactivity by this group to LPS by Ig of any isotype tested (seen in Fig 3) would support the notion the outliers with IgG responses to t1477 are convalescent cases. The IgM reactivity against t1477 reflected the IgA response. A notable exception was broadly similar levels of IgM reactivity by ‘*S*. Typhi’, and ‘No Growth’ groups. This contrasts with the highly significant difference seen when LPS-specific IgM was measured (Fig 3C). This appears to be caused by the reduced sensitivity for detection of typhoid by t1477-specific IgM, rather than any increase in sensitivity for detection of potential typhoid cases among the ‘No Growth’ group. As with LPS-specific IgM, t1477-specific IgM did not discriminate well between typhoid and ‘NTS’ groups, although again, this appears to be caused by the reduced sensitivity for detection of typhoid.

### Discrimination between groups by Receiver Operating Characteristics (ROC) analysis

**Fig 5:**
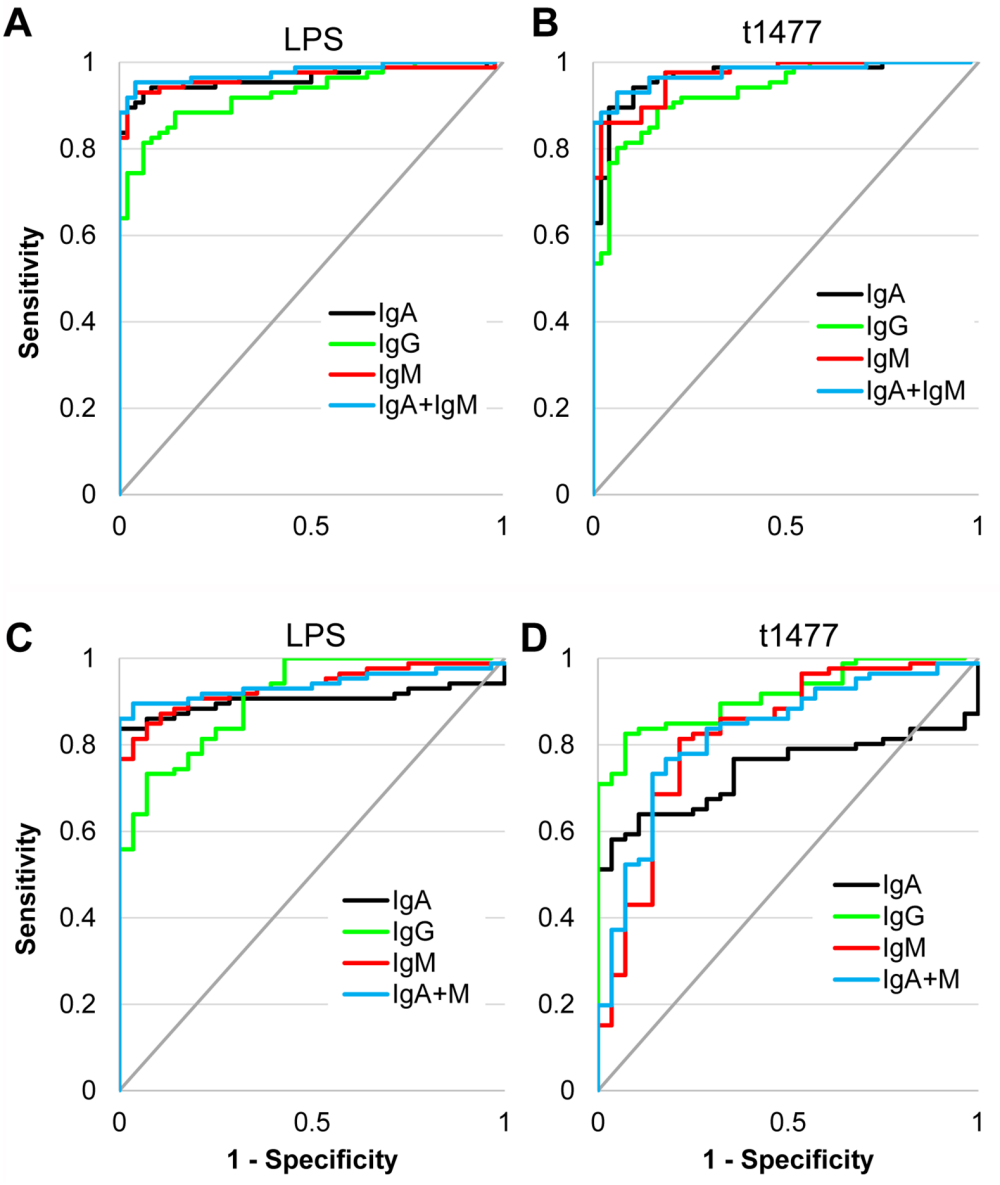
Discrimination between Nigerian pediatric typhoid and healthy controls or other febrile cases by LPS and HylE (t1477). (A) ROC plot of Nigerian pediatric typhoid (N = 86) vs. Nigerian pediatric healthy controls (N = 48) by LPS. (B) As panel A, instead using HylE. (C) ROC plot of Nigerian pediatric typhoid (N = 86) vs. Nigerian pediatric ‘other’ infections (N = 28) by LPS. (D) As panel C, instead using HylE. Corresponding values for % sensitivity, % specificity, and AUC are shown in Table 3 for panels A and B, and Table 4 for panels C and D. LPS, lipopolysaccharide; HylE, hemolysin E (t1477); ROC, receiver operator characteristic; AUC, area under the curve.

The accuracy of LPS and t1477 ELISAs to discriminate between Nigerian pediatric *S*. Typhi patients and controls were determined by ROC analysis. Plots of true positive rate (sensitivity) and false positive rate (1-specificity) for discriminating between typhoid cases and healthy children are shown for LPS and t1477 in Fig 5A and 5B, respectively. Table 3 shows corresponding percent specificity and sensitivity with either set at 90%, and areas under the curve (AUC). With LPS, IgA and IgM both gave 94% sensitivity (at fixed specificity) when used alone, which was increased slightly (to 95%) by combining the detection of IgA and IgM in the assay. Combining IgA and IgM could also be achieved *in silico* by summing the OD450nm data for IgA and IgM ELISAs performed individually (S4 Fig). LPS-specific IgA and IgM also give identical specificity when used alone (98% at fixed sensitivity) which was unchanged by combining both isotypes. The AUC of IgA and IgM were very similar (0.963 and 0.968, respectively) and increased slightly (0.978) after combining. Despite similar performance of IgA and IgM in the ROC analysis, the IgA ELISAs were characterized by lower backgrounds in the control groups compared to IgM, as can be seen from the raw data in S2 Fig. In contrast, LPS-specific IgG provided the lowest accuracy for distinguishing typhoid cases from healthy controls.

**Table 3:** ROC analysis of Nigerian *S*. Typhi (N = 86) vs. Nigerian healthy controls (N = 48).

Antigen	Isotype	Fixed specificity		Fixed sensitivity		AUC
		Sensitivity	Specificity	Sensitivity	Specificity	
**LPS**	IgA	94%	90%	90%	98%	0.963
	IgG	84%	90%	90%	71%	0.928
	IgM	94%	90%	90%	98%	0.968
	IgA+IgM	95%	90%	90%	98%	0.978
**t1477**	IgA	94%	90%	90%	96%	0.968
	IgG	81%	90%	90%	83%	0.932
	IgM	86%	90%	90%	88%	0.968
	IgA+IgM	93%	90%	90%	94%	0.976

Values correspond to ROC analysis shown in Fig 5A and 5B. Columns show % sensitivity and % specificity when either was fixed at 90%, and AUC. ROC, Receiver operator characteristic; AUC, area under the ROC curve.

In the t1477 ELISAs, although AUC values of IgA and IgM were identical (0.968), IgA provided superior sensitivity than IgM (94% and 86%, respectively, at fixed specificity) and specificity (96% and 88%, respectively, at fixed sensitivity). Multiplexing IgA and IgM did not increase sensitivity or specificity over IgA alone, although there was a modest increase in AUC (to 0.976). As with LPS, t1477-specific IgG also gave lower accuracy than IgA or IgM for diagnosing acute typhoid. These data indicate both LPS and t1477-specific IgA and IgM provide good discrimination between healthy Nigerian children and those with acute typhoid fever, which is improved by detection of both IgA and IgM isotypes together.

We then compared acute typhoid with 28 Nigerian ‘other’ (non-*Salmonella*) infections (listed in Table 2), since this is more relevant to the diagnosis of typhoid in the clinical setting. ROC Plots are shown in Fig 5C and 5D, with corresponding AUC, and percent sensitivity and specificity given in Table 4. Here, LPS-specific IgA and IgM give comparable sensitivity when used alone (86% and 87%, respectively, at fixed specificity), which is increased to 90% when IgA and IgM are combined. LPS-specific IgM provided considerably greater specificity than IgA when used alone (82% and 75%, respectively, at fixed sensitivity), which is dramatically increased (to 96%) when combined. The AUC is also increased slightly by combining IgA and IgM to 0.938. LPS-specific IgG provides the lowest sensitivity and specificity of all three isotypes. By comparison, the relative accuracy of t1477 in ELISAs for diagnosing acute typhoid was lower for all three Ig isotypes compared to LPS, and also reduced relative to discrimination of typhoid vs. healthy controls. Unexpectedly IgG emerged as the isotype with the highest sensitivity and specificity of t1477-specific Igs. It is possible this is restricted to childhood, where there is relatively less lifetime exposure to *Salmonella* than older children and adults, combined with a robust IgG response during typhoid fever.

**Table 4:** ROC analysis of Nigerian *S*. Typhi (N = 86) vs. Nigerian ‘other’ infections (N = 28).

Antigen	Isotype	Fixed specificity		Fixed sensitivity		AUC
		Sensitivity	Specificity	Sensitivity	Specificity	
**LPS**	IgA	86%	90%	90%	75%	0.903
	IgG	73%	90%	90%	68%	0.909
	IgM	87%	90%	90%	82%	0.934
	IgA+M	90%	90%	90%	96%	0.938
**t1477**	IgA	64%	90%	90%	0%	0.740
	IgG	84%	90%	90%	68%	0.914
	IgM	43%	90%	90%	46%	0.824
	IgA+M	53%	90%	90%	46%	0.826

Values correspond to ROC analysis shown in Fig 5C and 5D. Columns show % sensitivity and % specificity when either was fixed at 90%, and AUC. ROC, Receiver operator characteristic; AUC, area under the ROC curve.

**Fig 6:**
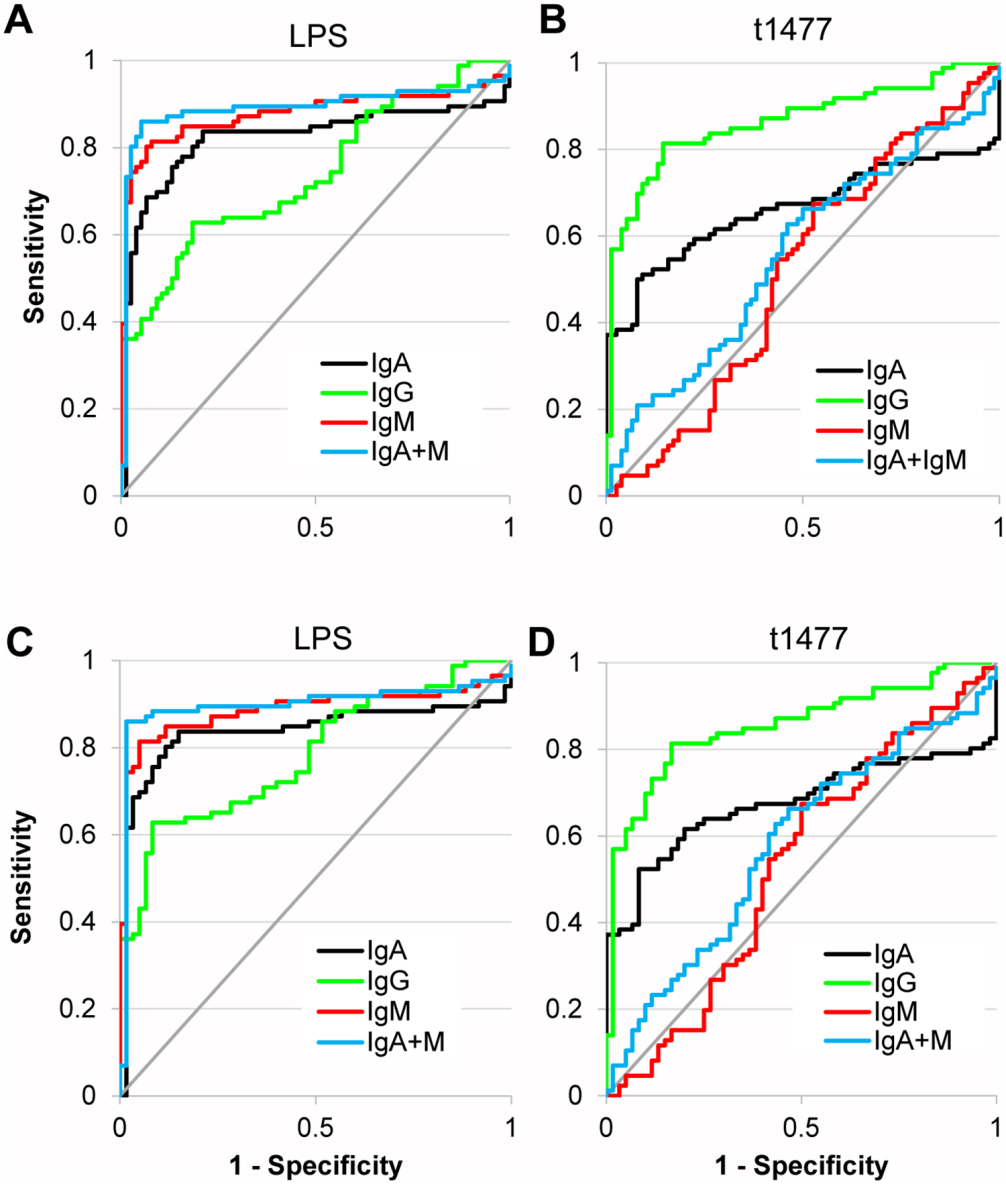
Discrimination between Nigerian pediatric typhoid and non-Nigerian other infections by LPS and HylE. (A) ROC plots of Nigerian pediatric typhoid (N = 86) vs. non-Nigerian “other” infections comprising Spanish tularemia (N = 12), Peruvian brucellosis (N = 16) and malaria from Kenya, Mali and Papua New Guinea (N = 49) by LPS. (B) As panel A, instead using HylE. (C) As panel A, except for removal of brucellosis cases. (D) As panel B, except for removal of brucellosis cases. Corresponding values for % sensitivity, % specificity, and AUC are shown in Table 5. LPS, lipopolysaccharide; HylE, hemolysin E (t1477); ROC, receiver operator characteristic; AUC, area under the curve.

We also compared the ability of LPS and t1477 to discriminate between Nigerian pediatric typhoid and additional samples from ‘other’ non-*Salmonella* infections obtained from locations outside Nigeria, namely tularemia (Spain, N = 12), brucellosis (Peru, N = 16) and malaria (various sources, N = 48). ROC plots are shown in Fig 6A (LPS) and 6B (t1477), with corresponding AUC and percent sensitivity and specificity given in Table 5. Data were broadly similar to that seen with Nigerian ‘other’ infections, with combined IgA+IgM providing the most accurate test when using LPS, and IgG providing the most accurate test when using t1477. As noted earlier, brucellosis samples were prominent among the ‘other’ infections for cross-reactivity to *S*. Typhi LPS. If these samples were removed from the analysis (Fig 6C and 6D) there was a slight increase in sensitivity and specificity in almost all situations, with the exception of t1477-specific IgG (Table 5, values in parenthesis).

**Table 5:** ROC analysis of Nigerian *S*. Typhi (N = 86) vs. non-Nigerian “other” infections combined (N = 76).

Antigen	Isotype	Fixed specificity		Fixed sensitivity		AUC
		Sensitivity	Specificity	Sensitivity	Specificity	
**LPS**	IgA	70(78)%	90%	90%	16(20)%	0.822(0.837)
	IgG	45(63)%	90%	90%	33(44)%	0.740(0.784)
	IgM	81(83)%	90%	90%	57(65)%	0.879(0.887)
	IgA+M	86(88)%	90%	90%	71(80)%	0.892(0.897)
**t1477**	IgA	51(52)%	90%	90%	0(0)%	0.663(0.676)
	IgG	72(70)%	90%	90%	54(48)%	0.865(0.855)
	IgM	7(5)%	90%	90%	14(17)%	0.516(0.531)
	IgA+M	21(21)%	90%	90%	4(5)%	0.559(0.579)

Values correspond to ROC analysis shown in Fig 6A and 6B, with values in parenthesis show values after removal of brucellosis samples from the analysis shown in Fig 6C and 6D. The non-Nigerian “other” infections comprise Spanish tularemia (N = 12), Peruvian brucellosis (N = 16) and malaria from Kenya, Mali and Papua New Guinea (N = 48). Columns show % sensitivity and % specificity when either was fixed at 90%, and AUC. ROC, Receiver operator characteristic; AUC, area under the ROC curve.

**Fig 7:**
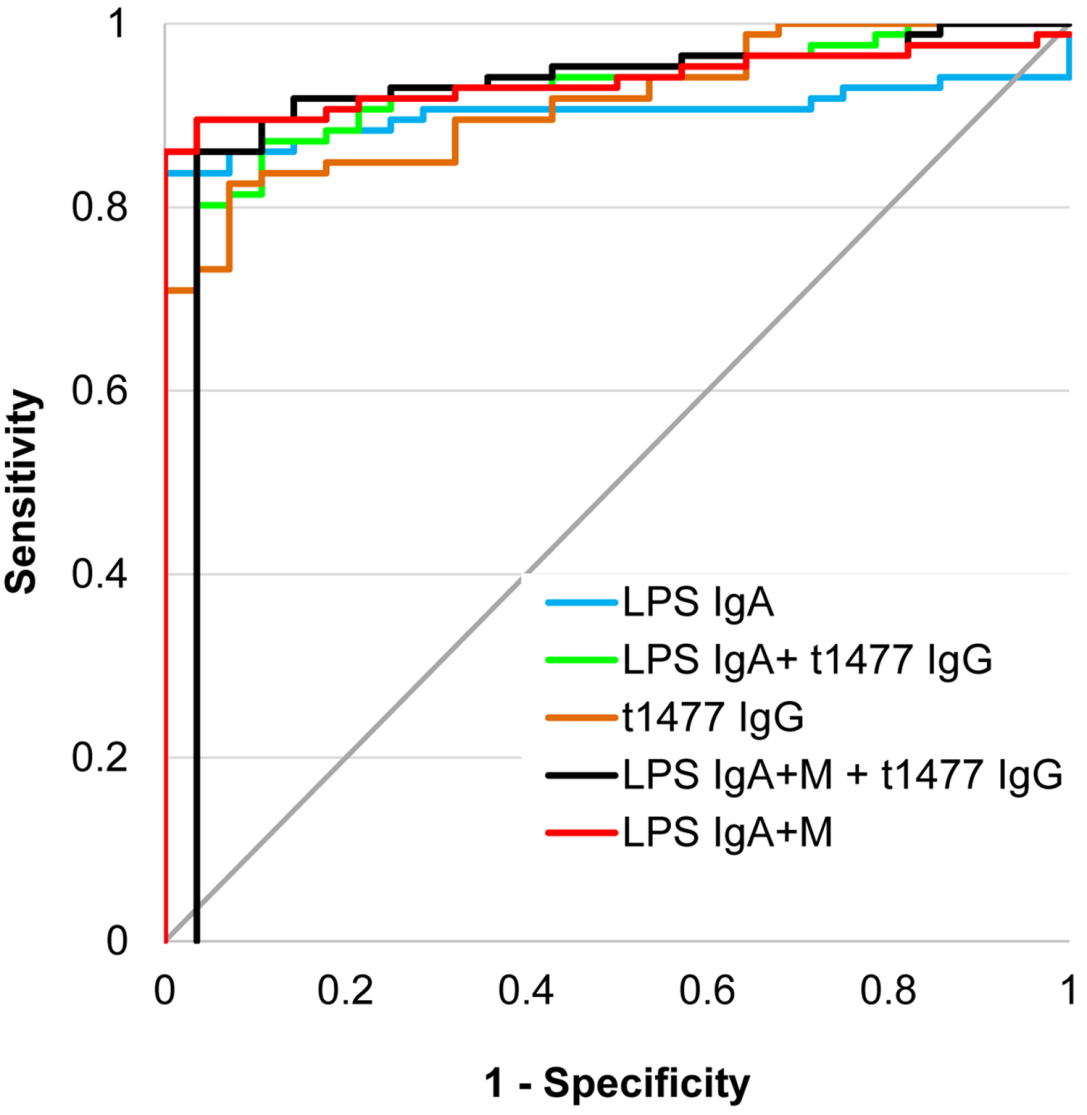
Discrimination between Nigerian pediatric typhoid and Nigerian other febrile cases by data multiplexed *in silico*. ROC plots of Nigerian typhoid patients (N = 86) vs. Nigerian ‘other’ infections (N = 28). Corresponding values for % sensitivity, % specificity and AUC are shown in Table 6. Antigens are ranked in ascending AUC. LPS, lipopolysaccharide; t1477, (hemolysin E, HylE); ROC, receiver operator characteristic; AUC, area under the curve.

Finally we explored the effect of multiplexing LPS and t1477 antigens on the accuracy of the test for typhoid compared to each antigen alone (Fig 7 and Table 6). Multiplexing LPS IgA with t1477IgG *in silico* increased accuracy compared to either alone, while multiplexing LPS IgA+IgM (as mixed secondary antibodies) with t1477 IgG *in silico* increased the accuracy further.

**Table 6:** Discrimination between Nigerian pediatric typhoid and Nigerian other febrile cases by data multiplexed in silico.

Antigen and Isotype	Fixed specificity		Fixed sensitivity		AUC
	Sensitivity	Specificity	Sensitivity	Specificity	
LPS IgA	86%	90%	90%	75%	0.903
LPS IgA+M	90%	90%	90%	96%	0.929
t1477 IgG	84%	90%	90%	68%	0.914
LPS IgA + t1477 IgG	87%	90%	90%	82%	0.939
LPS IgA+M + t1477 IgG	92%	90%	90%	93%	0.950

Values correspond to ROC analysis shown in Fig 7. Columns show % sensitivity and % specificity when either was fixed at 90%, and AUC. ROC, Receiver operator characteristic; AUC, area under the ROC curve.

## Discussion

In countries in Sub-Saharan Africa, where typhoid and non-typhoidal salmonellosis are major causes of bacterial sepsis in children, accurate and rapid point-of-care tests are urgently needed to replace existing diagnostic methods. Culture of *S*. Typhi organisms from bone marrow is the gold standard, but because it is invasive, blood culture is often a more practical, albeit less sensitive, alternative. Blood or bone marrow culture is also slow (2–3 days to arrive at a diagnosis), and empiric broad-spectrum antibiotic treatment is often initiated without a diagnosis being made.

The traditional Widal’s test, which is based on the agglutination of inactivated *Salmonella* Typhi and *Paratyphi A* organisms by antibodies to flagellin and LPS (H and O antigens, respectively) is rapid, inexpensive and requires no instrumentation. However, interpretation of the results must be made with caution. Sensitivity of the Widal’s test is lower in the early stage of infection when antibody titers are low. The test also fails to discriminate between acute from convalescent infection, leading to reduced sensitivity in endemic settings [20]. Although sensitivity can be improved if a follow-up sample is tested [21], this is not an option for rapid diagnosis. The test also lacks specificity owing to cross-reactivity with antibodies against closely-related NTS serovars [22] and other bacteria, notably *Brucella* [16]. Misuse of the Widal’s test has contributed to over-diagnosis of *Salmonella* infection, inappropriate antibiotic use, and the emergence of drug resistance [23].

Recent alternatives for serodiagnosis of typhoid include the Tubex test for LPS-specific IgM and the Typhidot test for IgG or IgM against a 50kDa outer membrane protein [24]. The Tubex test format is based on the interference by patient serum antibodies with the agglutination of latex beads coated with O9-specific monoclonal antibody and *S*. Typhi LPS-coated magnetic beads. The Typhidot test is a pre-dotted antigen strip. Neither test is currently configured for detection of IgA. Both have been evaluated in several Asian and African study sites; Tubex and Typhidot show comparable performance and were more specific although less sensitive than the Widal test (http://www.who.int/bulletin/volumes/89/9/11-087627/en/).

In this study we have focused on the use of LPS and t1477 (hemolysin E) as antigens to discriminate between *S*. Typhi infection and other bacterial infections, including commonly-encountered bacteremia seen in Nigeria. LPS has long been recognized as dominant in the response to *Salmonella*, while the identity of t1477 has come from studies using proteome-wide serological screens using microrrays [17, 25, 26]. Although the microarray has the potential to diagnose multiple infectious diseases on a single chip, it is currently unsuitable as a point-of-care test for many clinics in its current format. An accurate, more deployable test, particularly if configured into a format able to provide a result in <30 minutes, could help curb the inappropriate use of antibiotics and stem the rise in antibiotic resistance in Nigeria.

The data presented here indicate LPS-specific IgA (or IgA+M combined) discriminate well between Nigerian children with typhoid and healthy Nigerian children (AUC = 0.963 and 0.978, respectively; Table 3). More importantly for the clinical setting, LPS-specific IgA (or IgA+M combined) also discriminates between Nigerian children with typhoid and children with ‘other’ (non-*Salmonella*) infections (AUC = 0.903 and 0.938, respectively; Table 4). Similarly, discrimination between typhoid cases and healthy children using t1477-specific IgA (Table 3) was comparable to that obtained with LPS-specific IgA, although discrimination between typhoid and ‘other’ cases using t1477-specific IgA (Table 4) was far less accurate than for LPS-specific IgA. One possibility is proteins antigenically related to *S*. Typhi t1477 hemolysin E are found in one or more of the other bacterial infections represented in the collection (see Table 2). Such potential cross-reactivity would reduce the diagnostic utility of the antigen for typhoid.

LPS-specific IgG provided less accuracy for discriminating between Nigerian pediatric typhoid and healthy Nigerian children (Table 3), which was reduced further when discriminating typhoid with ‘other’ infections (Table 4). It is possible that IgG titers remain elevated for longer than IgA, thereby making it more difficult to discriminate between acute and previous or convalescent infections using IgG. This may be less of an issue in children where lifetime exposure to *Salmonella* species will likely be less than in adults. Although we have not examined Nigerian adults in this study, the expectation is they will have higher and more durable IgG titers to both LPS and t1477 than in children. This notion is supported for LPS by the pilot LPS array (Fig 2) in which all the non-Nigerian samples (i.e., panels E through K) were from adults. Thus, the median IgG signal of the healthy Nigerian children was lowest among all the groups tested, including adults from two non-endemic sites, the US (Fig 2K) and UK (Fig 2I). It remains to be determined whether LPS- and/or t1477-specific IgA has any utility for diagnosing typhoid in adults.

Unexpectedly, t1477-specific IgG performed better than LPS-specific IgG for discriminating between Nigerian pediatric typhoid and healthy Nigerian children (Table 3), and between Nigerian pediatric typhoid with ‘other’ infections (Table 4). Indeed, t1477-specific IgG also performed better than t1477-specific IgA and IgM for discriminating between typhoid and ‘other’ infections in Nigerian children. It is possible this diagnostic performance occurs only in children, where there is less lifetime exposure to *Salmonella*. It is anticipated that t1477-specific IgG will have less utility for diagnosing acute typhoid in older children and adults where the IgG titers from convalescent infections are likely to be much higher.

Finally we also compared the ability of LPS and t1477 to discriminate between typhoid and non-Nigerian ‘other’ infections from other locations around the world. In the ELISA, LPS-specific IgA+M provided excellent sensitivity and specificity, although we did notice detection of some Peruvian brucellosis cases using *S*. Typhi LPS (Fig 3 and Table 5). There are accounts in the literature of antigenic cross-reactivity between *Brucella* sp. and *S.* *enterica* serotype Urbana [16, 27, 28] which raises the possibility of cross-reactivity between antibodies generated during human brucellosis and *Salmonella* antigens. Although brucellosis is rare in Nigeria, if discrimination between acute typhoid and brucellosis is necessary, one option might be to utilize serodiagnostic *B*. *melitensis* antigens discovered previously [29–32] or *B*. *melitensis* LPS (Fig 1G) to assist in positive identification of brucellosis cases. The accuracy of t1477-specific IgA (or IgA+M) was lower than for LPS, consistent with its performance in discriminating between typhoid and Nigerian “other” infections.

LPS has received considerable interest as a potential diagnostic antigen for typhoid and for the basis of alternative assays to the Widal’s test. In one longitudinal study [33], anti-LPS IgA and IgM titers were seen to peak around d11-21 and decline thereafter, whereas IgG titers remained elevated and did not decline as rapidly. Other studies have also shown the transient nature of anti-LPS IgA in typhoid in saliva samples [34, 35] as well as in sera of gastroenteritis caused by non-typhoidal *Salmonella* serovars [36]. Thus, LPS-specific IgA appears to be a useful marker of acute Salmonellosis owing to its transient appearance after infection. The transient nature of IgA appears to be a peculiarity of LPS, and possibly other T-independent antigens, since serum and mucosal IgA responses to bacteria are generally long-lived [37–39].

In the present study, the LPS molecule did not discriminate well between typhoid and NTS, presumably because of the presence of shared epitopes present in the conserved lipid A and core oligosaccharide regions [40]. However, the more variant O-polysaccarides where serovar-specific epitopes of the O-antigen are located may discriminate between antibodies engendered by typhoid and NTS serovars. *Salmonella* O-polysaccharides have been produced from bacterial extracts and conjugated to protein carriers for use as subunit vaccines [41–43], although their utility as specific diagnostics is less well explored. In one such study, the *S*. Typhi O-polysaccharide O:1,9,12 performs well in IgG dot blots as a discriminator between typhoid and other acute infections or healthy controls, although IgA and the ability to discriminate between typhoid and NTS were not examined [44]. Neither the use of LPS nor measurement of IgA for diagnosis of typhoid is novel, but when used together appear to represent a good marker for acute infection in Nigerian children.

The t1477/hemolysin E (HylE, also known as cytolysin A or CylA) protein is a known dominant antigen in the antibody response to *S*. Typhi infection [17, 25, 26, 45]. Its utility as a potential serodiagnostic for typhoid has been demonstrated independently in a study of different Ig isotypes in 50 culture-confirmed typhoid cases [46]. In that study, IgA was the most sensitive, detecting 28/50 cases using a cut-off defined by the isotype-matched responses by healthy controls and other febrile infections. IgG was second most sensitive (19/50), and IgM least sensitive (3/50). A subsequent pilot study has demonstrated the utility of anti-HylE IgA in saliva as a biomarker for acute typhoid fever [47]. *S*. Typhi HylE is a 302 amino-acid long transmembrane protein with a helix hydrophobic segment located between residues 179 and 199. Along with homologs in other bacteria, such as the prototypic ClyA in *E*. *coli*, *S*. Typhi HylE belongs to a family of important pore-forming virulence factors of bacterial pathogens that assemble in cell membranes [48]. The HylE gene (t1477) is present in human-specific typhoid serovars (Typhi and Paratyphi) but absent from others (e.g., *S*. Typhimurium). In Fig 4, IgA reactivity by the 29 Nigerian children with invasive NTS (iNTS) is negligible with the exception of two outliers with low reactivity. However, sensitivity of t1477-specific IgA for detection of typhoid is also low, indicating this antigen is unlikely to have utility for discriminating iNTS and typhoid.

## Methods

### Ethics statement

This study was conducted with informed consent and approved by the Ethics Committees of the Federal Capital Territory of Nigeria, Federal Medical Center Keffi, Aminu Kano Teaching Hospital and University of Nebraska Medical Center (UNMC), Omaha Institutional Review Board (IRB). We used written consent provided by parent or guardian of each child. The process was approved by both local IRB and UNMC IRB.

Sera from Spanish tularemia cases were provided by Drs. Raquel Escudero and Pedro Anda, Instituto de Salud Carlos III, Madrid, Spain. Human subjects approval from Comité de Bioética y Bienestar Animal, Instituto de Salud Carlos III (approval no. PI 33). Sera from Thai melioidosis cases were provided by Direk Limmathurotsakul and Narisara Chantratita, Mahidol University, Thailand. Ethical approval for the study was from the Ministry of Public Health, Royal Government of Thailand, and the Oxford Tropical Research Ethics Committee. Sera from Peruvian brucellosis cases were collected with human subjects approval from the Human Research Protection Committee of the University of California San Diego, the Comite de Ética of Universidad Peruana Cayetano Heredia, Lima, Peru, and the Comite de Ética of Asociación Benéfica PRISMA, Lima, Peru. Sera from Bangladeshi cholera cases were provided by Drs. Edward Ryan, Richelle Charles and Firdausi Qadri, Massachusetts General Hospital, Boston, MA. Human subjects approval by IRB protocol # 1999P009116 and International Centre for Diarrhoeal Disease Research, Bangladesh (ICDDRB) #PR-11041. Sera from *Clostridium difficile* infections were collected with ethical approval from the University of Liverpool Research Ethics Committee (#08/H1005/32), and each patient provided written informed consent prior to recruitment. Malaria sera were collected with human subjects approvals from Institutional Review Boards at University Hospitals Case Medical Center and the Kenya Medical Research Institute Ethical Review Committee [49], the Medical Research Advisory Council, PNG [50], and the Ethics Committee of the Faculty of Medicine, Pharmacy, and Odonto-Stomatology and the Institutional Review Board at the National Institute of Allergy and Infectious Diseases, National Institutes of Health [51]. Sera from healthy US adults were collected under UCI IRB protocol #2007–5896. Sera were provided to the University of California Irvine (UCI) for assay without patient identifiers and were classified as exempt status by the UCI Institutional Review Board.

### Sera

A retrospective study was designed using a convenience series of sera samples from Nigerian pediatric febrile cases and healthy controls, as well as other infectious diseases from other locations outside Nigeria, which were assayed by ELISA and/or LPS microarray (Table 1). The Nigerian samples were collected between 2009 and 2014 from children aged 8 months—13 years (median approximately 4 years) who presented to primary or secondary health centers in central and northwest Nigeria with an acute febrile illness and other symptoms that were suggestive of bacteremia. The duration of symptoms ranged from about 3–10 days with a median of 5 days, as documented in the clinical data captured during enrollment. *S*. Typhi is the leading cause of childhood bacteremia in this area [52]. Baseline demographics of this population have been described previously [52, 53]. Following informed consent from the parent or guardian, blood was obtained aseptically from a peripheral vein for blood culture and simultaneously an aliquot for serum separation was saved. Blood sampling and processing were as previously described [52, 53]. Briefly, only aerobic blood culture bottles were used and held in a Bactec 9050 incubator (Becton Dickinson, Temse, Belgium) for a maximum of 5 days. Bacteria were identified by morphology, and for Enterobacteriacae, by use of an API 20 E rapid identification system (BioMerieux, Marcy-l'Étoile, France). Bacterial isolates were stored in skimmed milk at -70°C, and further characterized at the Clinical Microbiology laboratory, University of Nebraska Medical Center. Bacteremia was defined as the isolation of at least 1 noncontaminant bacteria from the admission blood culture. These samples comprised children with typhoid (N = 86), non-typhoid *Salmonella* (NTS) infections (N = 29), other bacteremias (N = 28), and febrile cases that were culture negative (‘No Growth’, N = 178). Samples sizes were determined by availability during the collection period. No samples with missing or indeterminate culture test results were used in this study. In addition, we also obtained sera from healthy Nigerian children enrolled from immunization clinics in the same facilities as controls (N = 48). These children present for routine immunizations and typically are in a stable state of health. Only children who were asymptomatic and did not have a history of a febrile illness in the past month, or had taken any antibiotic during the same period, were eligible. No blood cultures were performed on the healthy controls.

For the pilot LPS array (detailed below), an expanded collection of samples from “other” control infections from other countries were tested in addition to Nigerian samples discussed above, as follows. 1) Tularemia sera (N = 12) from a 2007 Spanish outbreak of *Francisella tularensis* subsp. *holarctica*. These consisted of paired samples from 6 acute cases that were seronegative by microagglutination (MA) test at the 1st time point at presentation and which seroconverted by the 2nd time point approximately 2 weeks later. These samples were found previously to be seropositive for *F*. *tularensis* subsp. *tularensis* (FTT) strain Schu S4 antigens at both time points using a proteome microarray [18]. 2) Melioidosis sera from Thailand (N = 14). Samples were collected in 2004 from patients presenting with symptoms of melioidosis, and were diagnosed by indirect hemagglutination assay (IHA) and blood and throat swab culture, as described previously [54]. 3) Brucellosis sera collected prior to 2008 from an endemic region of Peru (N = 28), previously shown to be seropositive using a *Brucella melitensis* proteome microarray [31, 32]. Samples probed were culture positive/Rose-Bengal positive (N = 12) and culture negative/Rose Bengal positive (N = 16). These correspond to samples taken on the first day (acute infection) and within 6 weeks after obtaining the first sample (convalescent infection).4) Cholera sera from Bangladesh collected between 2008 and 2010 presenting to the International Centre for Diarrhoeal Disease Research, Bangladesh (ICDDRB) hospital with acute watery and stool culture confirmed *V*. *cholerae* O1 infection. Following informed consent, venous blood was collected from adults (age 18–55 years) at the acute phase of infection (N = 7) after clinical stabilization (day 2), and again at convalescent phases of infection (d7 and 30; N = 7). 5) Sera from *Clostridium difficile* infections (CDI) from diagnosed acute cases in the UK collected between 2008 and 2012 (N = 16) [55]. Each patient was followed-up for minimum period of 30 days initially and then for 1 year from notes for the collection of additional demographics clinical outcome information. 6) Symptomatic malaria cases from Kenya, Papua New Guinea and Mali (N = 48). These were diagnosed with *Plasmodium falciparum* parasitemia, and all defined as seropositive using different iterations of *P*. *falciparum* protein arrays derived from strain 3D7 [56]. 7) Healthy US adults from a non-endemic area (Orange County, CA), Adherence to Standards for Reporting of Diagnostic Accuracy Studies (STARD) is shown by the flowchart (S5 Fig) and checklist (S1 Text) in the Supporting Information.

### LPS microarray

Lipopolysaccharides (LPS) were obtained as follows: 1) LPS from *S*. Typhosa (= *S*. Typhi) was purchased from Sigma-Aldrich (Cat. #L2387); 2) LPS from *S*. Typhimurium was purchased from Sigma-Aldrich (Cat. #L6511); 3) LPS from *Francisella tularensis* Subsp. *novicida* was purified from the live vaccine strain (LVS) (DSTL batch #B07/3564), as described [57]; 4) LPS from *Burkholderia pseudomallei* was purified from strain K96243 (DSTL batch #B07/3558), as described [58]; 5) LPS from *Brucella melitensis* was purified from strain 16M, as described [31]; 6) LPS from *V*. *cholerae* O1 was purified from Ogawa (strain X-25049) and Inaba (strain T19479) serotypes, as described [59]; 7) *Escherichia coli* 055:B5 LPS was purchased from Sigma-Aldrich (Cat. #L2880). Each LPS species was diluted in PBS buffer, pH 7.3–7.5 (EMD Millipore Corp., Billerica, MA; Cat. #6506-OP) and printed on nitrocellulose-coated glass slides (Oncyte Avid from Grace Bio-Labs, Bend, OR) using a GeneMachines Omnigrid 100 array printer, and printed at a concentration of 0.1 μg/ml. This concentration was determined previously in titration experiments to be the lowest concentration able to provide near maximal signals. Performers of the LPS microarray assays were blinded to the identity of the samples until after the assays were completed. LPS arrays were probed for 18h at 4°C with sera diluted 1/100 in protein microarray blocking buffer (Maine Manufacturing, GVS North America, Sanford, ME) supplemented with *E*. *coli* lysate (Antigen Discovery Inc, Irvine, CA). Bound IgG and IgA were then detected using secondary antibodies conjugated to biotin followed by streptavidin conjugated to quantum dots, and then visualized in an ArrayCAMarray imager, as described previously [19].

### ELISA

Hemolysin E protein (HylE, gene t1477 from *S*. Typhi Ty2 strain) was expressed in *E*. *coli* and purified as described previously [17]. LPS from S. Typhi was as described above for microarrays. ELISAs were performed as described [60]. Briefly, antigens were coated onto microtiter plates (ThermoScientific, Walham, MA) at concentrations 1.25 μg/ml (LPS) and 2.5 μg/ml (HylE) in TBS (100μl/well) overnight at 4°C. The coating concentrations were determined previously for each antigen by serial dilution experiments. The following day, plates were washed 4 times in 1x TBS containing 0.05% Tween20 (T-TBS; ThermoScientific) and blocked with casein/TBS blocking buffer (ThermoScientific) for 1-2h (300 μl/well). Blocking buffer was then decanted, and the plates air-dried and stored in desiccated foil pouches at 4°C until required for use. Performers of the ELISAs were blinded to the identity of the samples until after the assays were completed. For ELISA assay, sera were diluted to 1/200 (LPS) and 1/100 (HylE) in casein/TBS blocking buffer containing *E*. *coli* lysate (GenScript, Piscataway, NJ) at 1.5 mg/ml final concentration, and incubated for 30 min prior to placing into the plates. Plates were incubated for 45 min with gentle rocking at room temperature (RT). After washing with T-TBS goat anti-human IgG-, IgA- or IgM-HRP conjugates (Bethyl Laboratories, Inc., Montgomery, TX) diluted 1/25,000 (IgG) or 1/12,500 (IgA, IgM) in Guardian Stabilizer (ThermoScientific) were added to wells (100 μl /well) and incubated for 45 min at RT (100 μl/well). After washing with T-TBS, plates were developed by adding 100 μl/well SureBlueReserve TMB developer (Kirkegaard and Perry Laboratories, Inc., Gaithersburg, MD) for 10 min in the dark. Development was stopped by addition of 100 μl/well of 0.2M H_2_SO_4_ and OD read at 450 nm in a Multiskan FC plate reader.

### Statistical methods

ELISA data were collected at OD450nm and data were corrected by the positive control between runs. Dot plots and comparisons between medians of different groups using the Wilcoxon method, were produced in JMP (SAS Institute, Inc., Cary, NC, USA). Receiver operator characteristic (ROC) analyses were performed between patient groups for each antigen with a varying threshold cut off in the R statistical environment using ROCR. Plots of false positive vs. true positive plots were made, from which areas under the curve (AUC) and sensitivity and 1-specificity values were calculated for each antigen(s).

## Supporting information

S1 Fig Scatter plot comparison of IgG ELISA data using two different batches of hemolysin E antigen (HylE, t1477) each assayed using the same 349 Nigerian samples; r^2^ = 0.922 and slope = 1.05 using Spearmann’s rank correlation. (BMP)

S2 Fig Bar chart of LPS ELISA data; data provided in file “S2 DATA_ELISA”. (BMP)

S3 Fig Bar chart of hemolysin E (HylE, t1477) ELISA data; data provided in file “S2 DATA_ELISA”. (BMP)

S4 Fig Scatter plot of IgA+IgM multiplex ELISAs using Nigerian typhoid and healthy pediatric samples obtained by either combining secondary antibodies in the assay (“combo”, x-axis) or by combining data of IgA and IgM obtained individually (“in silico”, y-axis); data provided in file “S2 DATA_ELISA”. (BMP)

S5 Fig STARD flow chart. (BMP)

S1 Text STARD checklist. (DOCX)

S1 DATA_LPS_ARRAY LPS microarray data, Excel file. (XLSX)

S2 DATA_ELISA ELISA data, Excel file. (XLSX)
