# Supplementary figures and images for: Development of ELISAs for diagnosis of acute typhoid fever in Nigerian children
Source: PLoS Negl Trop Dis. 2017 Jun 22;11(6):e0005679. doi: 10.1371/journal.pntd.0005679 (PMC5498068; doi:10.1371/journal.pntd.0005679)

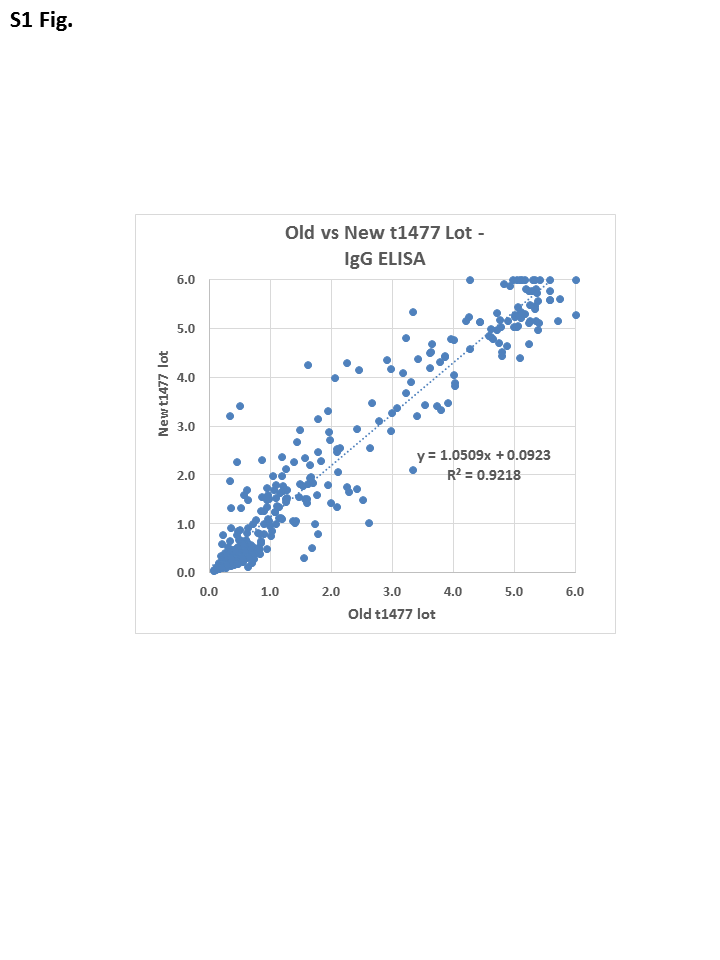

Supplement: S1 Fig — (BMP) [file pntd.0005679.s001.bmp]

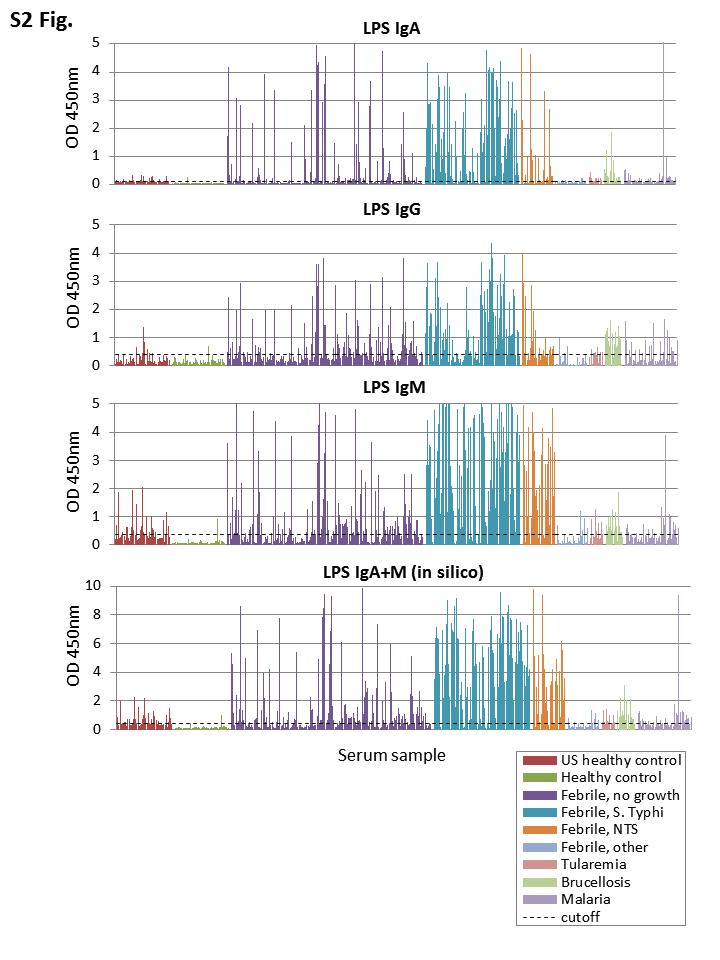

Supplement: S2 Fig — (BMP) [file pntd.0005679.s002.bmp]

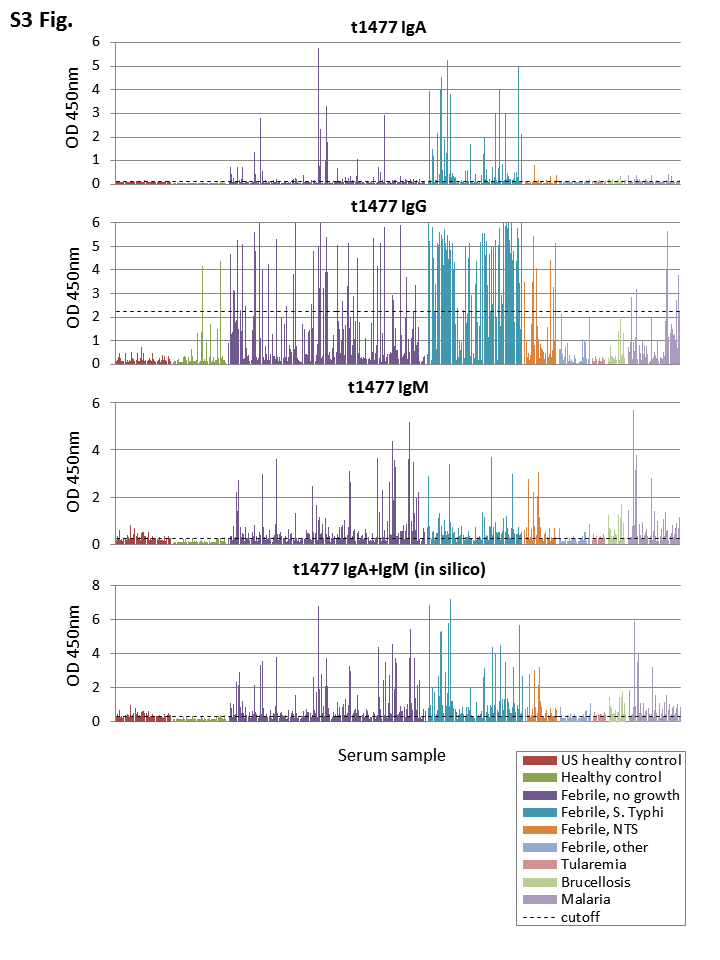

Supplement: S3 Fig — (BMP) [file pntd.0005679.s003.bmp]

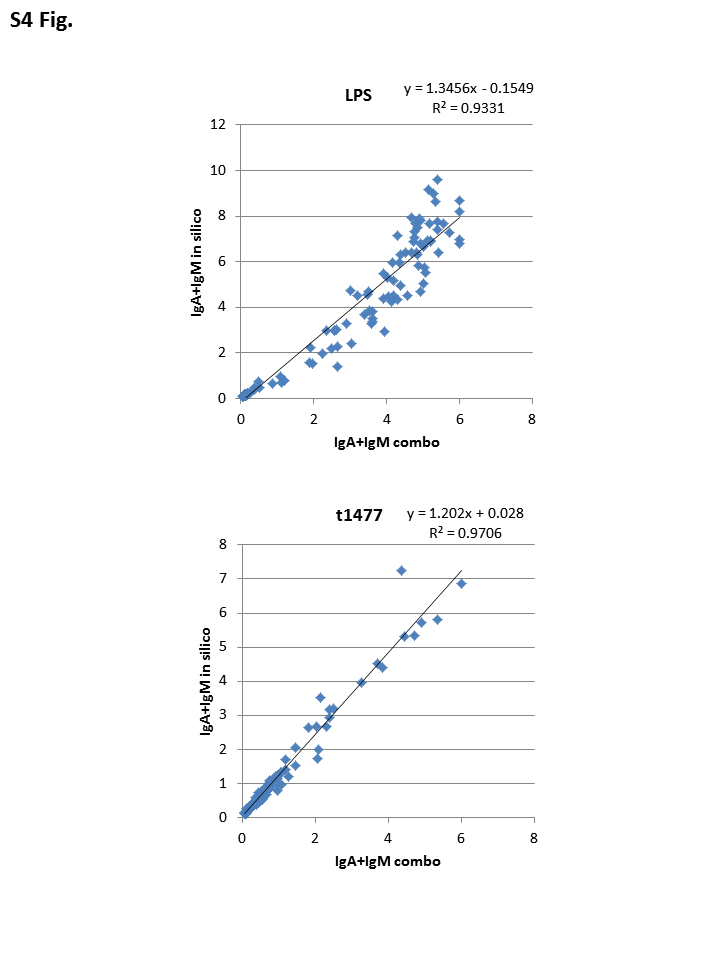

Supplement: S4 Fig — (BMP) [file pntd.0005679.s004.bmp]

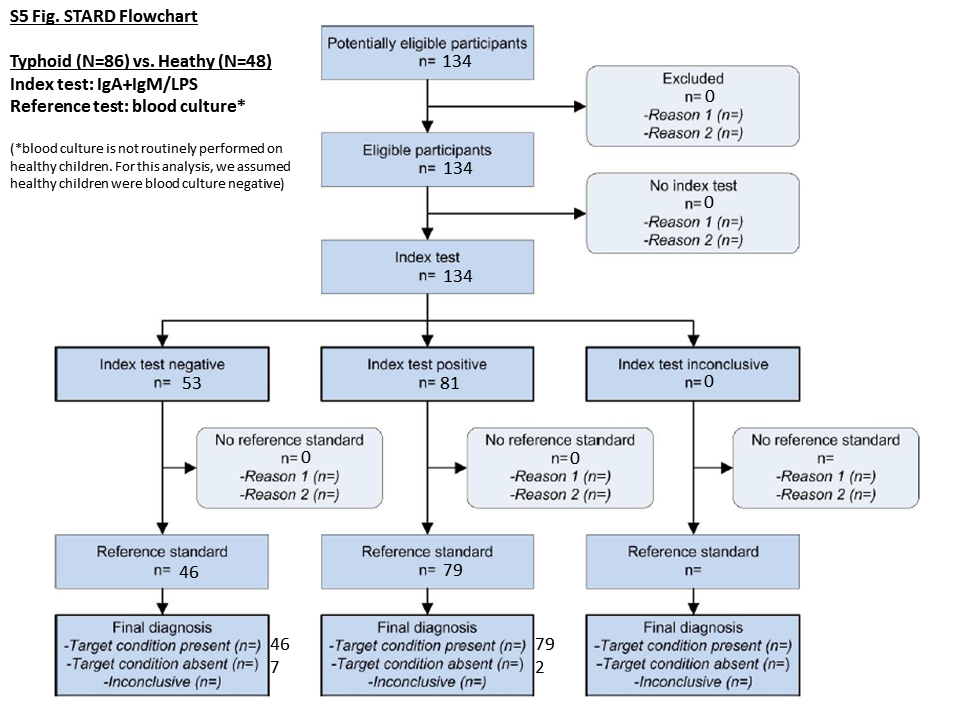

Supplement: S5 Fig — (BMP) [file pntd.0005679.s005.bmp]
